# Anomalous small-angle X-ray scattering analyses on hierarchical structures of rubber–filler systems

**DOI:** 10.1107/S1600576723000547

**Published:** 2023-03-09

**Authors:** Yuki Watanabe, Shotaro Nishitsuji, Mikkihito Takenaka

**Affiliations:** a Institute for Chemical Research, Kyoto University, Gokasho, Uji 611-0011, Japan; b Graduate School of Organic Materials science, Yamagata University, 4-3-16 Jonan, Yonezawa, Yamagata 992-8510, Japan; Lund University, Sweden/cny>; Keele University, United Kingdom

**Keywords:** anomalous small-angle X-ray scattering, ASAXS, hierarchical structures, inter-aggregate correlations

## Abstract

The hierarchical structures of poly(styrene-*ran*-butadiene) (SBR) rubber/carbon black (CB) systems vulcanized with sulfur and ZnO have been clarified using anomalous small-angle X-ray scattering (ASAXS) near the Zn absorption edge. In the case of SBR/CB systems vulcanized with peroxide, it has been found previously that the hierarchical structures formed by CB consist of aggregates of primary particles and agglomerates of those aggregates with mass-fractal dimensions. However, to date the hierarchical structures in SBR/CB systems vulcanized with sulfur and ZnO have not been well investigated, despite being commonly used. This is because the strong scattering contrast of Zn prevents the quantitative analyses of the hierarchical structures of CB using X-ray scattering. In this study, the effects of Zn on the scattering intensity were eliminated and the structure factors of CB in SBR/CB systems were obtained using the ASAXS method. By extrapolating to the zero volume fraction of CB, the particle structure factor of the CB aggregates was estimated and it was found that the CB aggregates consist of closely packed CB primary particles. The presence of large particles of ZnO and particles of ZnS on the order of 10 nm in size is confirmed.

## Abstract 2

In this work, the structure factors of carbon black (CB) and Zn were estimated independently in poly(styrene-*ran*-butadiene)/CB systems containing Zn using the anomalous small-angle X-ray scattering method. The particle structure factor obtained for CB revealed that the CB aggregates consist of close-packed CB primary particles. Large particles of ZnO and particles of ZnS were identified and found to be on the order of 10 nm in size.

## 1. Introduction

**Figure 1:**
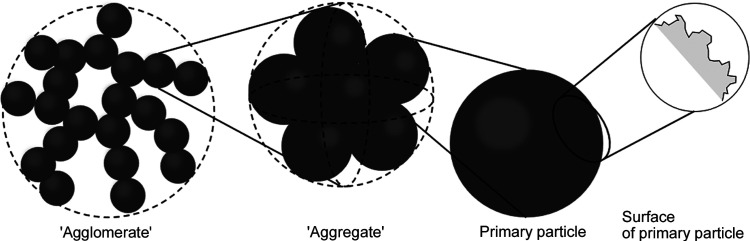
Schematic for hierarchical structures of filler in rubber.

Rubber reinforced by fillers such as silica and carbon black (CB) is widely used in our daily life. The mechanical properties of rubber–filler systems depend on the structures formed by the fillers. Thus structure analysis is critical in order for us to control their mechanical properties. Many researchers have investigated the structures of rubber–filler systems using scattering techniques (Baeza *et al.*, 2013 ▸; Koga *et al.*, 2008 ▸, 2005 ▸; McGlasson *et al.*, 2020 ▸; Noda *et al.*, 2016 ▸; Rishi *et al.*, 2018 ▸; Shinohara *et al.*, 2007 ▸; Takenaka *et al.*, 2009 ▸; Yamaguchi *et al.*, 2017 ▸) and reported that the fillers form hierarchical structures in rubber as shown in Fig. 1 ▸. Koga *et al.* (2008 ▸) found that primary particles of CB form aggregates on the order of 10 nm and that aggregates form agglomerates by connecting with mass-fractal dimensions in poly(styrene-*ran*-butadiene) (SBR)/CB systems.

However, many scattering experiments on rubber–filler systems use samples vulcanized with peroxide rather than with sulfur and zinc oxide (ZnO), even though vulcanization with sulfur and ZnO is common in practical use. This is because ZnO has a strong contrast factor in X-ray scattering. Though the amount of ZnO is usually less than 5% by volume in rubber–filler systems, the scattering intensity of ZnO in such systems is strong enough to affect the scattering intensity of conventional X-ray scattering. Thus, the effect of ZnO on the scattering intensity makes quantitative analyses of the hierarchical structures of fillers difficult. Moreover, the particle size of ZnO in rubber–filler systems is typically 100–1000 nm and is similar to the size of aggregates of fillers (Staropoli *et al.*, 2020 ▸; Morfin *et al.*, 2006 ▸). Thus, we need to eliminate the effects of ZnO from the scattering intensity to analyze the hierarchical structures of rubber–filler systems quantitatively.

To remove the effects of ZnO, we employed anomalous small-angle X-ray scattering (ASAXS). ASAXS is a contrast variation scattering technique that enables us to estimate the structure factors of each component in a multicomponent system (Lyon & Simon, 1987 ▸; Naudon, 1992 ▸; Stuhrmann, 2007 ▸; Hoell *et al.*, 2009 ▸). It is well known that the scattering length and contrast factor of X-rays drastically change near absorption edges (Cromer & Liberman, 1981 ▸; Sasaki, 1989 ▸). In the ASAXS experiment, we measured the incident X-ray energy dependence of the scattering intensity near the absorption edges of one component in the multicomponent system and obtained each structure factor of the system by analyzing the variation of the scattering intensity with the X-ray energy of the incident X-rays, as described later. In the case of rubber–filler systems with ZnO, the *K*-absorption edge of Zn is used for ASAXS. Morfin *et al.* (2006 ▸) applied ASAXS to estimate the structure factors of ZnO and filler separately using the *K*-absorption edge of Zn. However, they did not analyze the hierarchical structure of the filler or the structure of ZnO in detail.

In this work, we used the ASAXS method to study how the amount of CB affects the hierarchical structures in SBR/CB systems vulcanized with ZnO, as studied by McGlasson *et al.* (2020 ▸). We also investigated the spatial distribution of Zn in SBR/CB systems.

## 2. Experimental

### 2.1. Sample

We used SBR (Nipol 1502, ZEON Corporation) as the rubber. The CB (HAF class grade) used in this study was obtained from TOKAI CARBON Co. Ltd, Japan. The characteristics of SBR are listed in Table 1 ▸. We prepared nine SBR/CB samples with different CB compositions, as listed in Table 2 ▸. CB, stearic acid (StAc) and ZnO were compounded into SBR using a Banbury mixer for 280 s and then mixed with sulfur and accelerator using an open roll mill. Subsequently, we molded the samples at 160°C for 30 min to vulcanize them.

### 2.2. ASAXS measurements

We conducted ASAXS measurements with BL03XU at SPring-8 in Hyogo, Japan. The energies of the incident X-rays were set to 9.634, 9.639, 9.644, 9.647, 9.650, 9.652, 9.655 and 9.657 keV below the *K*-absorption edge of Zn. The PILATUS-1M was used as a two-dimensional detector. The sample-to-detector distances were 2.5 and 7.8 m, and thus the observed magnitude of the scattering vector *q* ranged from 0.012 to 1.8 nm^−1^ [*q* is defined by

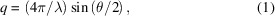

where λ and θ are the wavelength of the incident beam and the scattering angle, respectively]. The 2D data obtained were corrected for absorption of the sample, air scattering was subtracted, and the data were then converted to 1D SAXS data by circularly averaging. The resulting curves were converted to absolute scattering power by comparison with the scattering from a calibrated Lupolen standard (Pilz, 1969 ▸).

## 3. Results and discussion

### 3.1. SAXS profile

**Figure 2:**
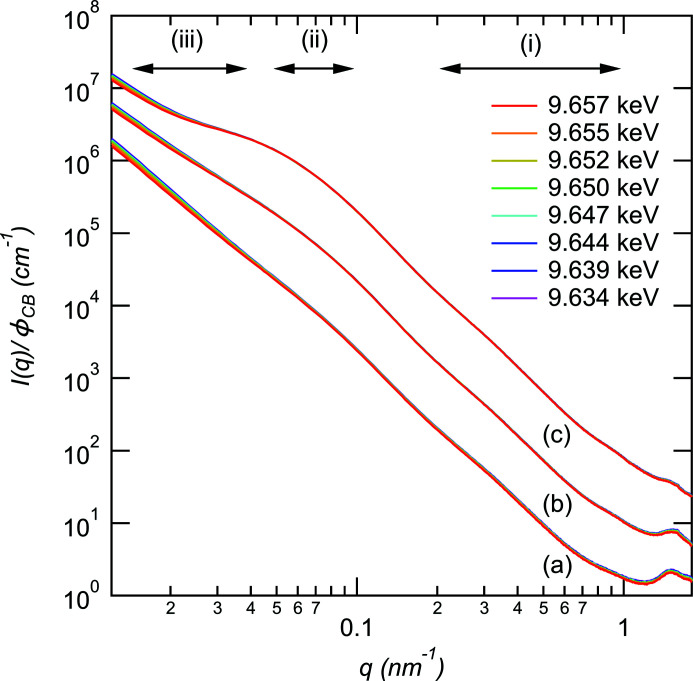
X-ray energy dependencies of the scattering intensity for (*a*) SBRCB05, (*b*) SBRCB20 and (*c*) SBRCB65. The scattering intensities for SBRCB20 and SBRCB65 are shifted vertically by factors of 10 and 100, respectively.

Fig. 2 ▸ shows the changes in the SAXS profiles for (*a*) SBRCB05, (*b*) SBRCB20 and (*c*) SBRCB65 with incident X-ray energy. According to previous studies by Koga *et al.* (2005 ▸), the scattering profiles of CB/SBR systems can be divided into the following several regions: (i) In the high-*q* region or 0.3 < *q* < 1 nm^−1^, the scattering profiles *I*(*q*) show power-law behaviors with an exponent of −3.3, reflecting the surface fractal of the CB surface. (ii) In the intermediate-*q* region or 0.04 < *q* < 0.1 nm^−1^, the shoulder characteristic of an aggregate consisting of primary particles is observed. (iii) In the lower-*q* region or *q* < 0.04 nm^−1^, the scattering intensity shows that the power-law behaviors originate from the network structures of the aggregates with mass-fractal dimensions. We found similar behavior in our systems, as shown in Fig. 2 ▸. In addition to the three characteristic features described above, we found a broad peak in the *q* > 1.0 nm^−1^ region for all samples. The origin of the peak is the Zn compounds, such as zinc stearate (ZnSt) (Salgueiro *et al.*, 2009 ▸, 2007 ▸; Yan *et al.*, 2014 ▸). The profiles of each sample change with X-ray energy at *q* < 0.03 nm^−1^ and *q* > 0.8 nm^−1^. At *q* < 0.03 nm^−1^, the structure or aggregation of ZnO on the submicrometre scale affects the scattering intensity. Thus, the shoulder becomes more distinct with increasing volume fraction of CB since the contribution of Zn decreases with increasing volume fraction of CB. As described above, the peak at 1.5 nm^−1^ originates from the Zn compound, in agreement with the fact that the profiles around *q* = 1.5 nm^−1^ vary with X-ray energy.

We analyzed the scattering profiles using the contrast variation method. The system is assumed to consist of three components, SBR, CB and Zn, and the scattering profiles can therefore be described as follows under the condition of incompressibility:

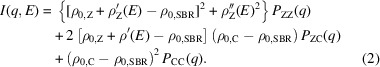

Here *P_ij_* (*q*) is the partial scattering function defined by

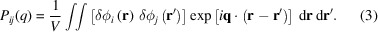


*V* is the scattering volume radiated by the incident beam and 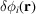 is the fluctuation of the volume fraction of component *i* at position 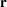 , where *i* and *j* are C (CB), Z (Zn) or SBR (SBR). ρ_0,*i*_ is the scattering length density of the *i*th component, defined by

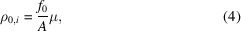

where *f*_0_ is the atomic scattering factor, *A* is the molar mass and μ is the specific gravity. Since we conducted SAXS experiments near the Zn absorption *K* edge, the scattering length density ρ_Z_ of Zn varies with the energy *E* of the incident X-rays and is expressed by

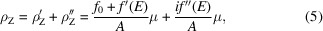

where *f*′(*E*) and *f*′′(*E*) are the real and imaginary parts of the anomalous dispersion, respectively. Under the condition where the fraction of Zn is much smaller than those of SBR and CB, we can neglect the contribution of *P*_ZC_(*q*) (Morfin *et al.*, 2006 ▸; Cenedese *et al.*, 1984 ▸). Thus, equation (2) can be simplified to

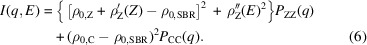

We obtained the vector of the scattering intensities 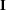 = [*I*(*q*, *E*_1_), *I*(*q*, *E*_2_), *I*(*q*, *E*_3_), *I*(*q*, *E*_4_), *I*(*q*, *E*_5_), *I*(*q*, *E*_6_), *I*(*q*, *E*_7_), *I*(*q*, *E*_8_)] from the SAXS experiments at each energy in all samples. 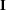 can be expressed by

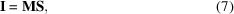

where **M** is the matrix of the difference of the scattering length density and 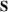 is the vector of partial scattering functions [*P*_ZZ_(*q*), *P*_CC_(*q*)]. **M** is expressed by

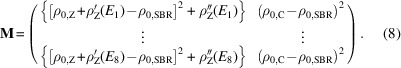

To decompose the scattering intensities into partial scattering functions, we need to calculate the transposed matrix **M** ^T^ satisfying 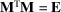 by singular value decomposition. By applying **M** ^T^ to 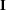 , 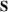 can be obtained using

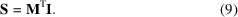

**Figure 3:**
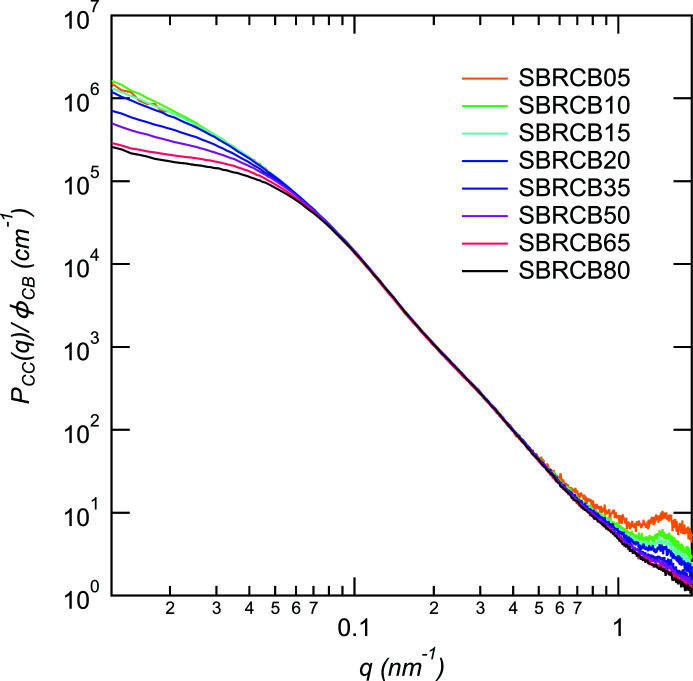
Partial scattering function *P*_CC_(*q*)/ϕ_CB_ of CB for all samples.

Fig. 3 ▸ shows the partial scattering function *P*_CC_(*q*)/ϕ_CB_ of CB. The shoulders of *P*_CC_(*q*) around *q* = 0.05 nm^−1^ become more distinct than those shown in Fig. 1 ▸, indicating that the lower-*q* region is affected by the scattering of ZnO. The position of the shoulder characteristic of the state of the aggregates shifts to higher *q* with increasing volume fraction of CB, indicating that the effects of inter-particle correlation between aggregates on *P*_CC_(*q*) increases with ϕ_CB_ (McGlasson *et al.*, 2020 ▸, 2019 ▸; Rishi *et al.*, 2018 ▸). On the other hand, the * q* dependence of *P*_CC_(*q*) at 0.1 < *q* < 0.5 nm^−1^ did not change with the volume fraction of CB, implying that the state of CB aggregation is independent of the volume fraction of CB. We observed a peak around *q* = 1.5 nm^−1^. This peak position agrees with the long period of the bilayer crystalline structure of ZnSt formed during vulcanization. The peak of ZnSt is observed even in *P*_CC_(*q*) because the electron density of the crystalline state of the St part in Zn is higher than that of the matrix SBR. The difference in the electron density between St and SBR can be attributed to the small amount of CB so that the peak appears in *P*_CC_(*q*).

**Figure 4:**
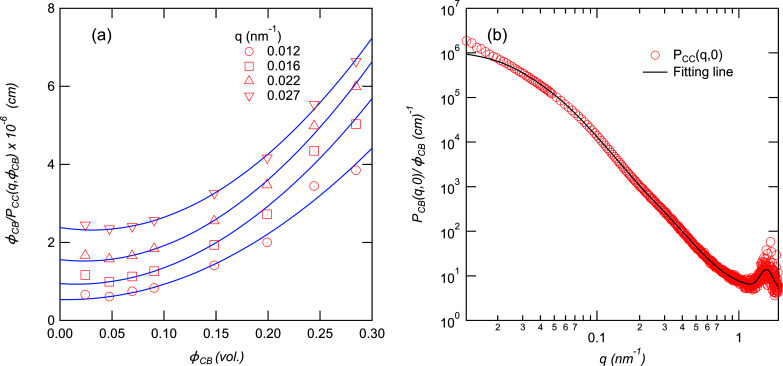
ϕ_CB_/*P*_CC_(*q*, ϕ_CB_) is plotted as a function of ϕ_CB_ at a given *q*. The legend shows the corresponding *q* value. Solid lines correspond to the fitting results with equation (10). (*b*) *P*_CC_(*q*, 0) is plotted as a function of *q* in a double logarithmic plot. The solid line corresponds to the fitting results with equation (12).

*P*_CC_(*q*) in the lower-*q* region decreases with increasing volume fraction of CB, suggesting that the inter-particle correlation increases with increasing volume fraction of CB. To estimate the particle scattering of the CB aggregates, we employed the following virial expansion to extrapolate to zero:

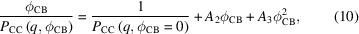

where *A*_2_ and *A*_3_ are second and third virial coefficients. Fig. 4 ▸(*a*) shows the ϕ_CB_ dependence of ϕ_CB_/*P*_CC_(*q*, ϕ_CB_) at a given *q*. We obtained *P*_CC_(*q*, ϕ_CB_ = 0) by extrapolating the data using equation (10). The *P*_CC_(*q*, ϕ_CB_ = 0) obtained is shown in Fig. 4 ▸(*b*).

The upturn found in the low-*q* region originates from the large CB particles >1 µm, as mentioned by Rishi *et al.* (2018 ▸). We analyzed *P*_CC_(*q*, 0) of the structures of the CB aggregates in the SBR/CB systems quantitatively using the following unified Guinier/power-law equation:

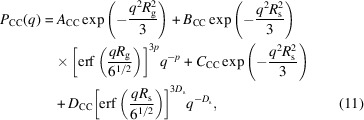

where *R*_g_, *R*_s_, *p* and *D*_s_ are the radius of gyration of aggregates, the radius of gyration of the primary particle, the surface-fractal dimension of the aggregate and the surface-fractal dimension of the primary particle, respectively. As described above, *P*_CC_(*q*) includes the scattering of the crystalline structures of ZnSt. Thus, we added a Gaussian function expressing the peak of ZnSt to equation (11) and fitted *P*_CC_(*q*, 0) with the following equation to characterize the aggregates of CB:

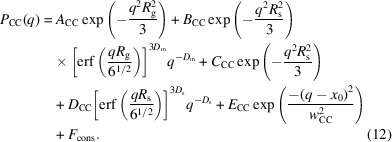


*A*_CC_, *B*_CC_, *C*_CC_ and *D*_CC_ are expressed by

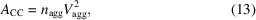




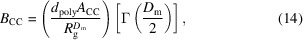




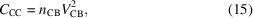




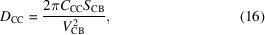

where *n*_agg_, *V*_agg_, *n*_CB_, *V*_CB_, *S*_CB_ and *d*_poly_ are the number of aggregates per unit volume, the volume per aggregate, the number of CB particles per unit volume, the volume per CB particle, the surface area per CB particle and the intrinsic dimension characterizing the degree of branching of the CB aggregation, respectively. *F*_cons_ is the contribution of the thermal diffuse scattering (Rathje & Ruland, 1976 ▸). The fitting results are shown in Fig. 4 ▸(*b*). We were able to fit the data with equation (12) and obtain the characteristic parameters as listed in Table 3 ▸.

Let us analyze the structure of the aggregates of CB particles. According to equations (13) and (15) and the volume fraction of CB particles, we can estimate the number of primary particles of CB in a CB aggregate *V*_agg_/*V*_CB_ using

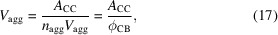

and

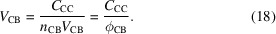

Thus,

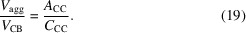

Substituting *A*_CC_ and *C*_CC_ into equation (12), we roughly estimated that there are 1.6 × 10^2^ primary particles in an aggregate, though the error bar of *C*_CC_ is too large to estimate the correct value. This value is almost the same as 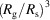 , indicating that the aggregates are composed mainly of CB.

To elucidate the change in the inter-aggregate correlation with the volume fraction of CB, here we assume that the scattering intensity can be described by the product of the structure factor *S*(*q*) and particle scattering function. The structure factor is defined by

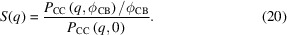

McGlasson *et al.* (2020 ▸) proposed a method to evaluate the inter-aggregate correlation. They used the random phase approximation to reveal the correlation between aggregates for CB. However, we used the polydisperse Born–Green approximation because the correlation between aggregates is expected to be stronger in a high volume fraction of CB. The structure factor is then given as

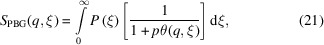




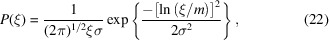




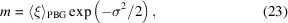




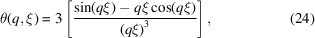

where *p* is the packing factor, θ(*q*, ξ) is the structure amplitude of the spherical scattering function, *P*(ξ) is distribution function between domains of aggregates, 〈ξ〉_PBG_ is the average distance between domains and σ is the distribution of correlation distances.

**Figure 5:**
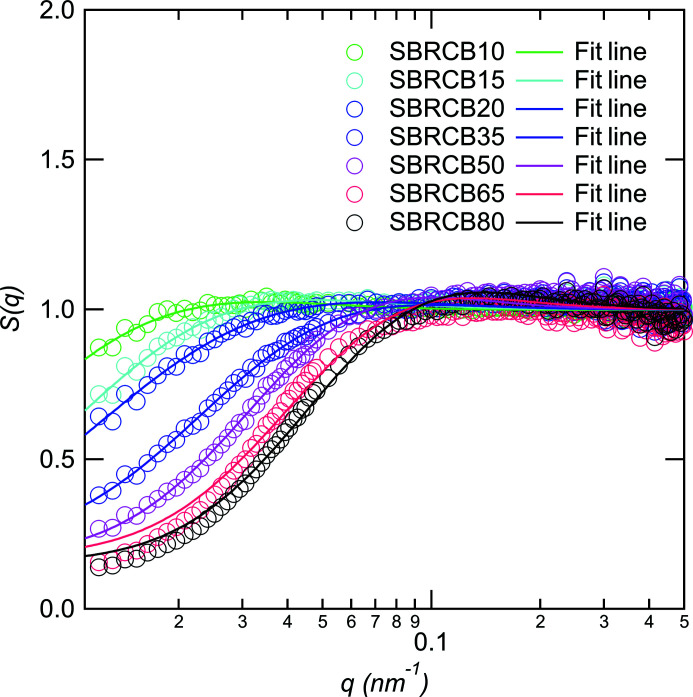
*S*(*q*) versus *q* for various CB volume fractions. The solid lines are the fitting results with equation (16).

**Figure 6:**
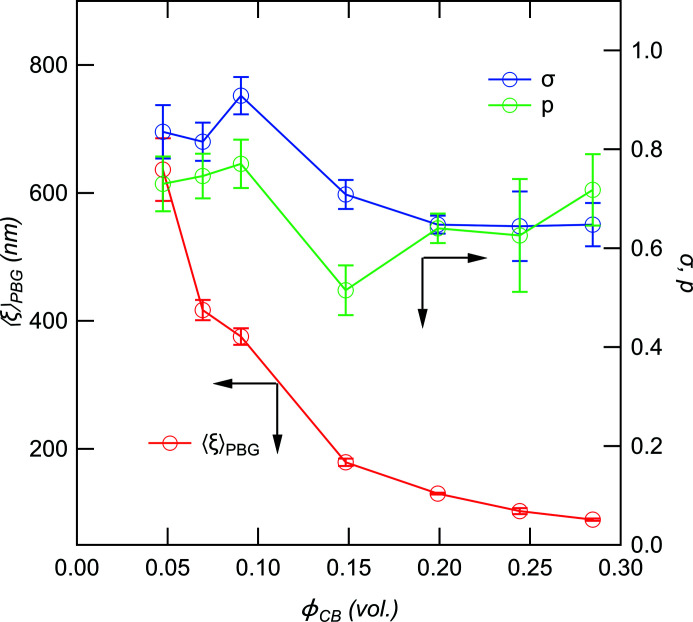
〈ξ〉_PBG_, *p* and σ are plotted as a function of ϕ_CB_.

Fig. 5 ▸ shows the structure factor *S*(*q*) versus *q* for various volume fractions of CB. Similar to the study by McGlasson *et al.* (2020 ▸), *S*(*q*) is almost one in the high-*q* region. This indicates that the change in the volume fraction of CB does not affect the particle scattering function. *S*(*q*) at *q* < 0.1 nm^−1^ decreases with increasing volume fraction of CB, reflecting the increase of inter-aggregate correlation with increasing volume fraction of CB. We were able to fit the data using equation (16), with the exception of the data for ϕ_CB_ = 0.02 (5 phr). The ϕ_CB_ dependencies of 〈ξ〉_PBG_, *p* and σ are shown in Fig. 6 ▸. 〈ξ〉_PBG_, *p* and σ decrease with increasing ϕ_CB_. We found the change of decay rate of 〈ξ〉_PBG_ to be between 0.09 and 0.148, whereas gradual changes can be found in the ϕ_CB_ dependencies of *p* and σ. The change in 〈ξ〉_PBG_ agrees with the results of the electrical percolation threshold by volume conductivity in the SBR/CB system (Janzen, 1975 ▸; Klueppel, 2003 ▸), reflecting the percolation threshold of CB aggregation (Isono & Aoyama, 2013 ▸; Kato *et al.*, 2006 ▸).

**Figure 7:**
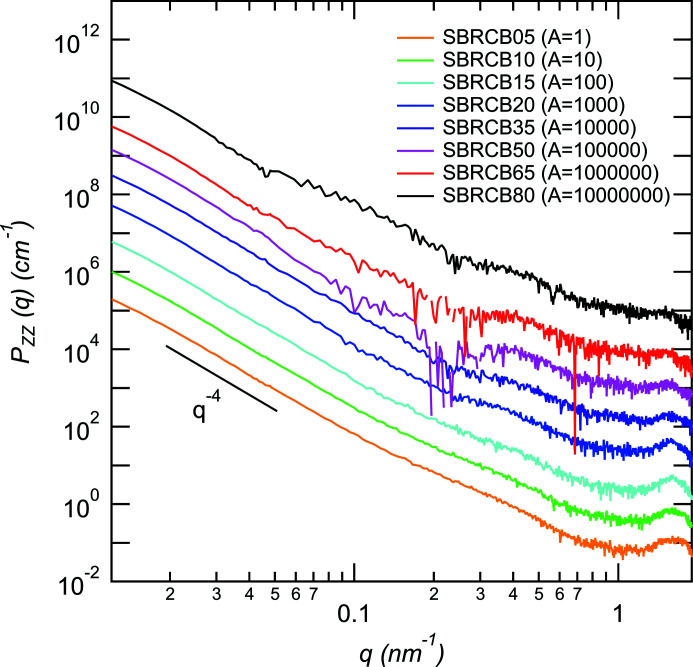
Partial scattering function of Zn for all samples. The functions are shifted by factors of *A* vertically.

Fig. 7 ▸ shows the partial scattering function of Zn. In the low-*q* region (0.01 < *q* < 0.1 nm^−1^), *P*_ZZ_(*q*) shows a power-law behavior with the exponent −4, reflecting the surface of the large particles of ZnO. Since we cannot observe the Guinier behavior for the ZnO particles in the observed *q* region, there are ZnO particles with radii >200 nm. In the high-*q* region, we observed a shoulder at around 0.2 nm^−1^ in addition to the peak corresponding to ZnSt at *q* = 1.5 nm^−1^. The shoulder corresponds to the ZnS particle formed during vulcanization (Kishimoto *et al.*, 2021 ▸; Shirode *et al.*, 2015 ▸). We used the 1-level unified Guinier power-law approach equation to estimate the size of ZnS:

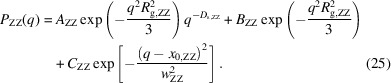

**Figure 8:**
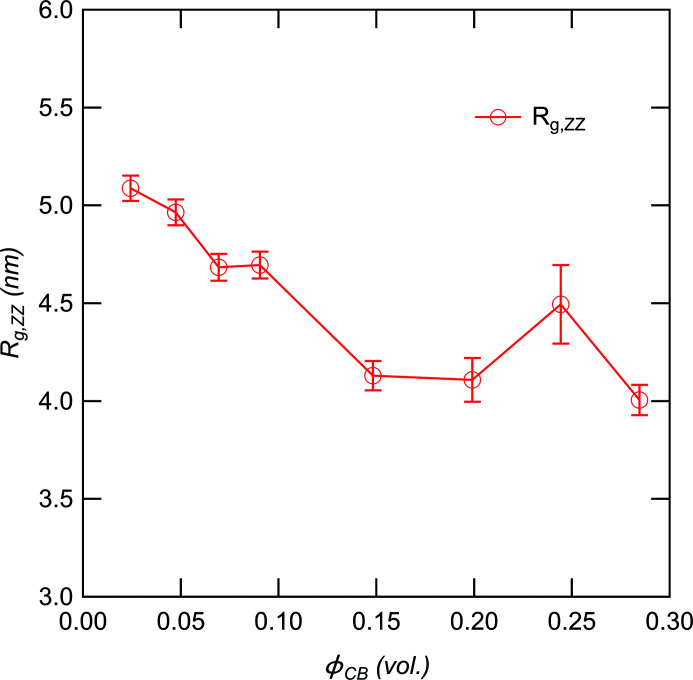
*R*_g,ZZ_ of ZnS plotted as a function of ϕ_CB_.

Here, *A*_ZZ_, *B*_ZZ_ and *C*_ZZ_ are the contrast prefactors, *D*_s,ZZ_ is the power law exponent, and *R*_g,ZZ_ is the radius of gyration of ZnS. Fig. 8 ▸ shows the ϕ_CB_ dependence of *R*_g,ZZ_. If the shape of ZnS is spherical, the size is about 10 nm and almost independent of ϕ_CB_, agreeing with the results of elemental mapping in rubber by energy-filtering transmission electron microscopy and SAXS (Dohi & Horiuchi, 2007 ▸). This result suggests that the vulcanization is not greatly affected by the presence of CB.

## 4. Conclusions

We applied the ASAXS method to SBR/CB systems vulcanized with sulfur and ZnO to investigate their hierarchical structures. We successfully eliminated the effects of Zn on the scattering intensity and obtained the structure factors of CB in SBR/CB systems using the ASAXS method. We measured the CB volume fraction dependence of the structure factor, and estimated the particle structure factor of the CB aggregate by extrapolating to the zero-volume fraction of CB. The CB aggregates are found to consist of closely packed CB primary particles. We also confirmed the presence of large particles of ZnO and particles of ZnS on the order of 10 nm.

## Figures and Tables

**Table 1:** Characterization of SBR *w*_PS_(%) – weight fraction of styrene content; vinyl content (%) – vinyl content in butadiene sequence.

Polymer	*M*_w_	*M*_w_/*M*_n_	*w*_PS_ (%)	Vinyl content (%)
SBR	5.0 × 10^5^	3.4	23.5	15

**Table 2:** Composition of samples (parts per hundred rubber, phr) used in this study Stearic acid – CH_3_(CH_2_)_16_COOH; accelerator – *N*-*tert*-butyl-2-benzo­thia­zyl sulfen­amide.

Code	SBR	CB	Stearic acid	Sulfur	Accelerator	Antioxidant	ZnO
SBRCB05	100	5	2.0	2.0	2.5	1.0	3.0
SBRCB10	100	10	2.0	2.0	2.5	1.0	3.0
SBRCB15	100	15	2.0	2.0	2.5	1.0	3.0
SBRCB20	100	20	2.0	2.0	2.5	1.0	3.0
SBRCB35	100	35	2.0	2.0	2.5	1.0	3.0
SBRCB50	100	50	2.0	2.0	2.5	1.0	3.0
SBRCB65	100	65	2.0	2.0	2.5	1.0	3.0
SBRCB80	100	80	2.0	2.0	2.5	1.0	3.0

**Table:** 

*A*_CC_	*B*_CC_	*C*_CC_	*D*_CC_	*E*_CC_
1.15 × 10^6^ ± 4.25 × 10^5^	12.3 ± 14.3	7.19 × 10^3^ ± 5.99 × 10^3^	2.79 ± 0.74	8.32 ± 0.46

**Table:** 

*R*_g_ (nm)	*D*_m_	*R*_s_ (nm)	*D*_s_	
67.7 ± 18	3.17 ± 0.43	14.0 ± 3.42	3.86 ± 0.18	

**Table:** 

*x*_0_ (nm^−1^)	*W*_CC_	*F*_cons_		
1.57 ± 0.01	1.99 × 10^−1^ ± 1.3×10^−2^	4.92 ± 0.40		
